# Metabolomic networks and pathways associated with feed efficiency and related-traits in Duroc and Landrace pigs

**DOI:** 10.1038/s41598-019-57182-4

**Published:** 2020-01-14

**Authors:** Victor Adriano Okstoft Carmelo, Priyanka Banerjee, Wellison Jarles da Silva Diniz, Haja N. Kadarmideen

**Affiliations:** 1 Quantitative Genomics, Bioinformatics and Computational Biology, Department of Applied Mathematics and Computer Science, Technical University of Denmark, Kongens Lyngby, Denmark; 2 Department of Genetics and Evolution, Federal University of São Carlos, São Carlos, Brazil

**Keywords:** Genetics, Animal breeding

## Abstract

Improving feed efficiency (FE) is a major goal of pig breeding, reducing production costs and providing sustainability to the pig industry. Reliable predictors for FE could assist pig producers. We carried out untargeted blood metabolite profiling in uncastrated males from Danbred Duroc (n = 59) and Danbred Landrace (n = 50) pigs at the beginning and end of a FE testing phase to identify biomarkers and biological processes underlying FE and related traits. By applying linear modeling and clustering analyses coupled with WGCNA framework, we identified 102 and 73 relevant metabolites in Duroc and Landrace based on two sampling time points. Among them, choline and pyridoxamine were hub metabolites in Duroc in early testing phase, while, acetoacetate, cholesterol sulfate, xanthine, and deoxyuridine were identified in the end of testing. In Landrace, cholesterol sulfate, thiamine, L-methionine, chenodeoxycholate were identified at early testing phase, while, D-glutamate, pyridoxamine, deoxycytidine, and L-2-aminoadipate were found at the end of testing. Validation of these results in larger populations could establish FE prediction using metabolomics biomarkers. We conclude that it is possible to identify a link between blood metabolite profiles and FE. These results could lead to improved nutrient utilization, reduced production costs, and increased FE.

## Introduction

With the expanding human population and requirement for nutrient-rich food, there is an increasing demand for improvement of meat production, but simultaneously, to decrease the input costs in terms of feed^1^. Thus, feed efficiency (FE) is the most important trait in commercial pig farming^2^ as increasing the amount of meat produced per feed is beneficial both economically and environmentally. Thereby, improving FE is beneficial for producers and increases the sustainability of pork meat production. Fortunately, FE is a highly heritable trait in Danish pigs (ranging from 0.34 in Duroc to 0.40 in Landrace), thus suitable for the genetic selection of pigs with high breeding values in breeding programs aimed at improving this economically important phenotype^3^.

Since FE cannot be measured directly, feed conversion ratio (FCR) and residual feed intake (RFI) have been used to evaluate the animal efficiency^4^. FCR determines the ratio of feed intake (FI) to output and found to correlate with growth rate and body weight^3,5^. RFI calculates the difference between the actual and expected FI^6^ predicted based on production traits such as average daily gain (ADG)^7^. ADG is also considered important in commercial pig production as pigs with higher ADG can achieve a target market weight within a shorter period than those with lower ADG, thereby saving feeding costs^8^. Thus, selection for RFI has proved to be effective in improving the FE in pigs^3,9,10^. Selection for FCR will results in co-selection for other traits, such as body composition and ADG. In contrast, RFI selects for increased metabolic efficiency without the same side effects^11–13^. RFI and FCR are well correlated, with a reported correlation of over 0.7 in the literature^3^.

As part of the existing genetic determinants of FE, genome-wide association studies (GWAS) and differential expression (DE) analyses have reported a large number of polymorphism and genes for RFI or FCR in pigs^9,14^. However, despite these efforts, FE is a complex trait with many aspects involved and the functional molecular background is still somewhat elusive^1^. Among the approaches, the metabolomics profile reveals the relationship between animal genetics and physiological phenotypes^15^, thereby increasing the fundamental understanding of efficiency and selection. Although affected by prandial activity, many metabolic processes underlie the transport of molecules through the blood. Blood is the sole way of absorption of nutrients into the body, and the blood metabolites are useful as a prime candidate for the study of FE in livestock^16^. In this context, it is also generally considered than improvement of RFI is associated with improved efficiency in the utilization of feed^11,12^ and thus improved utilization of nutrients.

An effective way to get insights into the interactions at a molecular level involved in complex phenotypes can be done by applying a network-based approach like weighted gene co-expression network analysis (WGCNA)^17^. In the context of metabolomics, the clusters (modules) represent specific metabolic processes or pathways and gives a better understanding of the function, interaction, and common regulatory mechanisms. WGCNA has been widely applied in pigs and several livestock species with fruitful results^18–21^. Therefore, one of the main objectives was to identify key blood metabolites associated with FE and related traits in Danbred Duroc and Danbred Landrace (referred to as Duroc and Landrace, respectively, further in the text). As the Durocs are more FE than the Landrace, the two breeds serve biological contrast in FE. Furthermore, selecting two diverse breeds can help generalize any results obtained versus only focusing on one breed.

Here, we applied an untargeted metabolomics approach for a better understanding of changes at a molecular level associated with nutrient utilization. We test the hypothesis that we are able to associate metabolite concentrations in blood at an early growth stage to predict future growth and FE measurements, and that metabolites profiles in general are associated with growth and efficiency phenotypes. We applied linear regression models to select the top metabolites predictive of FE, combined the results from network-based methods, and conducted a functional enrichment and pathway analyses to provide potential easy-to-screen candidate metabolite biomarkers and metabolic processes modulating FE in pigs.

## Results

### Descriptive statistics and linear model analysis

The phenotypic traits summary, including feed consumed (FC), FE, daily gain (DG), and delta weight (DW), for 109 pigs from Duroc and Landrace breed is shown in Supplementary Table S1. Aiming to ascertain the metabolite profiles concerning FE, we collected the blood samples at two time points (start and end of testing phase) from two breeds of pigs, profiled for the metabolite changes. The start phase was labeled as time point 1 (TP1) and the breeds as Duroc 1 and Landrace 1, while the end of the testing phase as time point 2 (TP2), mentioned as Duroc 2 and Landrace 2.

The number of metabolites for each of the breeds at each time point with p-value ≤ 0.05 are provided in Table 1. The molecular mass, retention time, and p-values of these metabolites for each trait in the breeds at different time points and at the combined time points are provided in Supplementary Table S2.

**Table 1:** Overview of metabolites associated with phenotypic traits in Duroc and Landrace at two time points.

Breeds^*^		FE	EDG	TDG	DG	RFI
Duroc 1	P ≤ 0.05^**^	62 (30)	42 (24)	33 (23)	47 (32)	64 (31)
	KS test	1.00E-05	0.19099	0.02317	0.02625	0
	FDR ≤ 0.05^***^	1	0	1	0	0
Duroc 2	P ≤ 0.05^**^	82 (40)	41 (26)	115 (68)	46 (26)	57 (28)
	KS test	0	0.10092	0	0.8561	0.02687
	FDR ≤ 0.05^***^	0	0	35	0	0
Landrace 1	P ≤ 0.05^**^	40 (17)	59 (37)	44 (22)	67 (38)	41 (16)
	KS test	0.25416	2.00E-05	0.00079	0	0.08764
	FDR ≤ 0.05^***^	0	9	0	1	0
Landrace 2	P ≤ 0.05^**^	54 (35)	37 (21)	77 (44)	73 (46)	53 (36)
	KS test	0	0	0	0	0
	FDR ≤ 0.05^***^	0	0	0	0	0
Duroc 1,2	P ≤ 0.05**	83 (46)	24 (12)	54 (23)	96 (61)	69 (36)
	KS test	0	0.004	7e-05	0	0
	FDR ≤ 0.05***	0	0	1	20	0
Landrace 1,2	P ≤ 0.05**	59 (35)	76 (40)	73 (37)	69 (34)	46 (29)
	KS test	0.06419	0	0	0	0.16896
	FDR ≤ 0.05***	0	0	0	0	0

*Numbers (1, 2) represents time point 1 and 2 respectively; ^**^Number of significant metabolites with p-value ≤ 0.05 (in the parenthesis are the number of annotated metabolites); P = p-value; KS test = Kolmogorov-Smirnov test; ^***^Number of metabolites with False discovery rate (FDR) ≤ 0.05; FDR = False discovery rate; FE = Feed efficiency; EDG = Early daily gain; TDG = Testing daily gain; DG = Daily gain; RFI = Residual feed intake.

**Figure 1:**
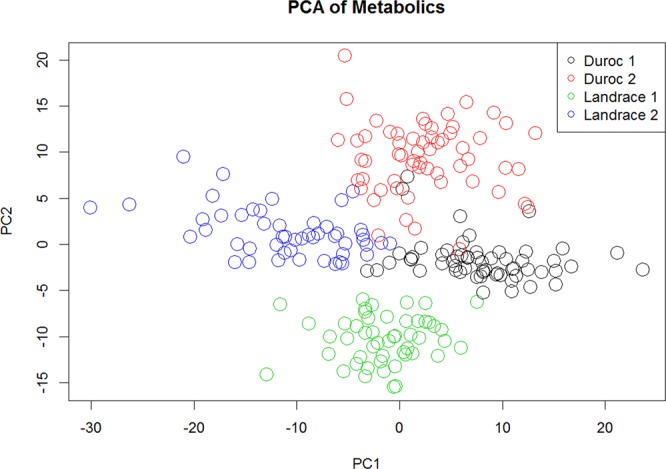
PCA visualization of the two first principal components, colored by breed and sampling. The first component separates the most divergent group – Duroc 2 and Landrace 1. The second component separates Duroc 1 and Landrace 2, the second most divergent group.

With an initial dataset of 729 metabolites, only those metabolites with relative standard deviation >0.15 were used for each group based on the raw counts. This amounts to 691 and 702 metabolites in Duroc (TP1 and TP2), while 684 and 689 for Landrace (TP1 and TP2), which were subjected for further analysis. To test if the metabolite profile was associated with the most distinct factors such as age and breed, the data were visualized on the first two principal components, colored by time point and breed, as given in Fig. 1. Further, the significance of the linear relationship for each metabolite between the two time points is observed (Supplementary Fig. S1). Over half of the metabolites have a p-value < 0.05 for the linear relationship between the two sampling points, indicating that there is a stability and predictability in the relative metabolite concentrations over time. In Fig. S2 we can see further evidence for this, with a visualization of all the pairwise log metabolite concentrations between the two time points, showing a clear overall relationship.

The significant metabolites at two time points were identified, as given in Table 1. A linear model was fitted to unravel the effect of blood metabolite on the FE phenotypes. The overall significance of divergence from the null hypothesis of no relation between metabolites and phenotypes using the Kolmogorov-Smirnov (KS) test, comparing the observed p-value distributions with the corresponding uniform distribution was tested. This was done to reveal, if there was an overall relation between the metabolites and our phenotypes. Most of the traits have significant metabolite profiles based on the KS test, signifying an overall relation between metabolites and traits. Based on the overall distribution of the KS test p-values, even the highest value of 0.19 in early daily gain (EDG) for Duroc could be significant based on FDR. In Duroc and Landrace, some metabolites were significantly associated with every trait, with the highest number identified in TDG (Table 1). The most significant results for testing daily gain (TDG) (35) and EDG (9) in Duroc 2 and Landrace 1, respectively, after false discovery rate (FDR) correction (Table 1) was identified.

**Figure 2:**
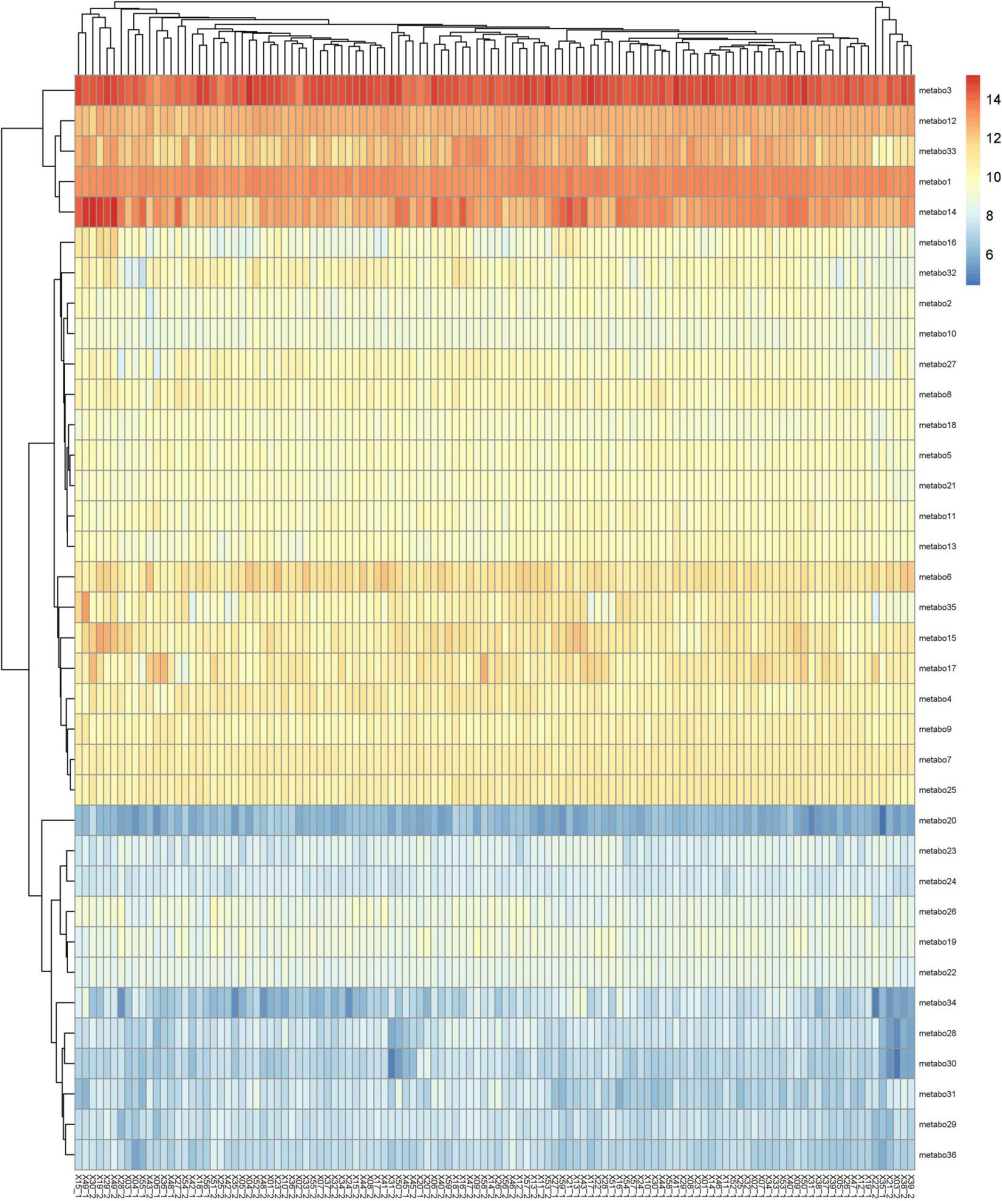
Heatmap constructed using the significant metabolites with RFI in Duroc (time point 1 and 2). The x-axis represents the sample ID at time point 1 and 2 represented as ID_1 and ID_2, respectively; the y-axis represents the metabolites (names of the corresponding metabolites are given in Supplementary Table S5.

**Figure 3:**
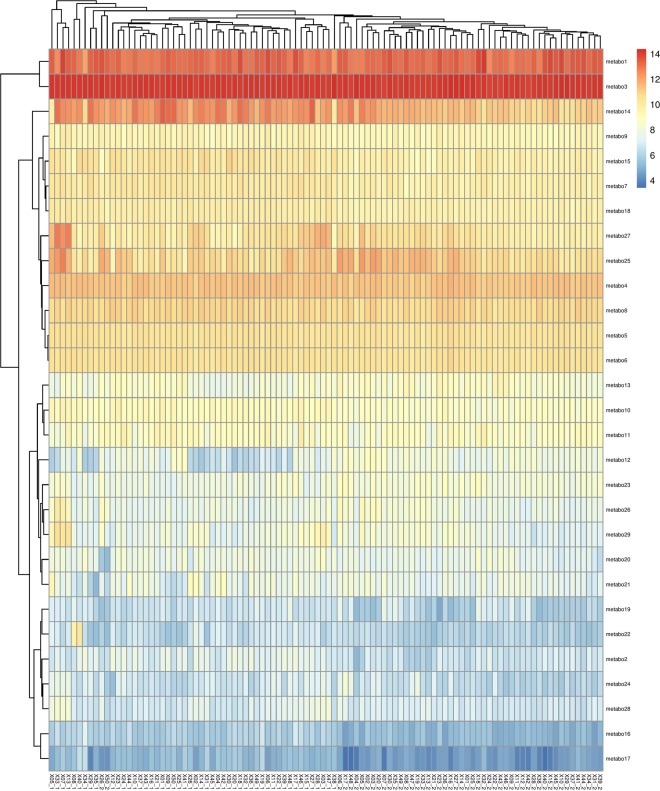
Heatmap constructed using the significant metabolites with RFI in Landrace (time point 1 and 2). The x-axis represents the sample ID at time point 1 and 2 represented as ID_1 and ID_2, respectively; the y-axis represents the metabolites (names of the corresponding metabolites are given in Supplementary Table S5.

We also did exploratory clustering analysis was done for the metabolites found significant for RFI in Duroc (36) and Landrace (29) (both time points combined) (Table 1, Supplementary Table S2). The heatmap plots in Duroc (Fig. 2) and Landrace (Fig. 3) grouped the metabolites in four specific clusters (Supplementary Table S5) and also the samples separately at TP1 and 2.

### Metabolite network analysis

Since the metabolites interact and/or are a part of the same or related metabolic pathways, a weighted gene network approach using WGCNA^17^, that is typically used for gene co-expression analyses, was adapted and implemented for metabolomics data. A signed weighted metabolite network was constructed following the WGCNA pipeline, which identifies modules of functionally related metabolites, summarizes the module based on module eigengene - ME, and relates the MEs with the trait of interest^17^. We constructed the networks separately for both the breeds at two time points to unravel the correlated metabolites with the trait of interest (FE, EDG, TDG, DG, and RFI). Next, we selected the significantly associated modules (p ≤ 0.1, and correlation ≥0.2) that were labeled by color for further analysis. The expression of any FE trait, such as RFI, is dependent on the stage of maturity while for other traits, this correlation is low^22,23^. However, in our study, we observed low to medium correlation for all the traits with respect to the metabolites.

**Figure 4:**
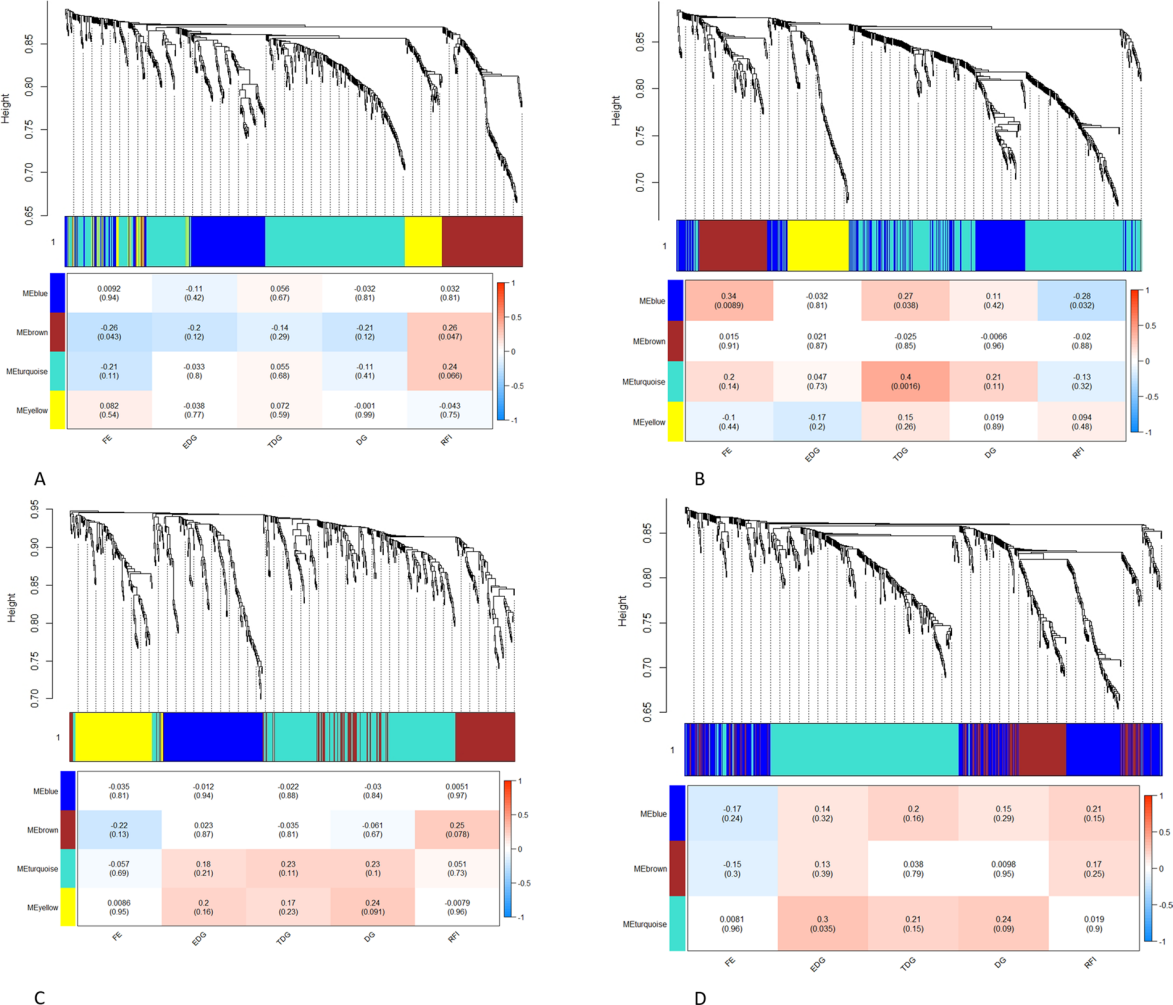
Clustering dendrogram and module-trait correlation plots. The upper panel of each plot (**A**–**D**) represents metabolite-clustering dendrogram obtained by hierarchical clustering of TOM-based dissimilarity with the corresponding module colors indicated by the color row. Each colored row represents color-coded module that contains a group of highly connected metabolites. The lower panel of each plot (a–d) represents the module trait correlation where the x-axis represents feed efficiency trait, and the y-axis represents the modules. Plots (**A**) and (**B**) represents Duroc at time points one and two, respectively, while plots (**C**) and (**D**) represent Landrace at time points one and two. The color-coding in the module-trait correlation plots is based on Spearman’s correlation (p-values in parenthesis). Positive and negative correlations are shown in red and blue colors, respectively.

In Duroc (TP1), 144, 131, 335, and 81 metabolites were clustered, respectively, in MEblue, MEbrown, MEturquoise, and MEyellow (Fig. 4A- upper panel). Among the modules, MEbrown was significantly associated with FE and RFI, and MEturquoise with RFI (Fig. 4A – lower panel). From the TP2, 190, 104, 316, and 92 metabolites were clustered in MEblue, MEbrown, MEturquoise, and MEyellow, respectively (Fig. 4B – upper panel). From these modules, significant associations were identified for MEblue (FE, TDG, and RFI), and MEturquoise (TDG) (Fig. 4B – lower panel). In Landrace (TP1), 152 metabolites were clustered in MEblue, 151 in MEbrown, 260 in MEturquoise while 121 in MEyellow (Fig. 4C – upper panel). MEbrown was significantly associated with RFI, while MEturquoise and MEyellow with DG at TP1 in Landrace (Fig. 4C – lower panel). Regarding TP2, 253 metabolites were clustered in MEblue, 142 with MEbrown and 294 with MEturquoise (Fig. 4D – upper panel). Nonetheless, only MEturquoise was associated with EDG and DG (Fig. 4D – lower panel).

The annotated metabolites with p ≤ 0.05 (Table 1, Supplementary Table S2) and those clustered into the associated modules (Fig. 4, Supplementary Table S3) were subjected to pathway over-representation analysis (Table 2). As the same metabolite in a module can be related to more than one trait, the unique metabolites were screened by taking all the significant modules for all the traits in each breed at each time point (Table 2). Then, we also screened the metabolites for commonality in each breed between the two time points. In Duroc, only a single metabolite out of 102 was found to be common between the two time points (TP1 and TP2). In Landrace, 36 metabolites were found common in two time points, while 66 metabolites were different in TP1 and TP2.

**Table 2:** Significant metabolites for FE traits used for pathway analysis.

Breed (Time point)*	Number of metabolites**	Module	Trait	Unique metabolites
Duroc (1)	11	Brown	FE	**32**
	9	Brown	RFI	
	21	Turquoise	RFI	
Duroc (2)	13	Blue	FE	**70**
	8	Blue	TDG	
	8	Blue	RFI	
	55	Turquoise	TDG	
Landrace (1)	8	Brown	RFI	**36**
	22	Turquoise	DG	
	6	Yellow	DG	
Landrace (2)	19	Turquoise	EDG	**37**
	36	Turquoise	DG	

*Numbers (1, 2) represents time point 1 and 2 respectively. **The metabolites were identified based on the overlapping of the linear model and network association modules. FE = Feed efficiency; EDG = Early daily gain; TDG = Testing daily gain; DG = Daily gain; RFI = Residual feed intake

### Pathway over-representation analysis

Exploiting the fact that metabolites are linked through biochemical reactions and thus are partaking in many pathways, we carried out a pathway over-representation analysis based on the integrated molecular level pathway analysis (IMPaLA) software^24^. To reveal the differences at the pathway level, we analyzed the unique metabolites in two different ways. First, by comparing the difference in the metabolites at two time points within the breeds. Second, by comparing the metabolite differences among the breeds (Duroc *vs*. Landrace), taking all the metabolites together irrespective of the time points in each breed (Table 2, Supplementary Table S3). The unique metabolites from two time points were screened, supporting the fact that the FI or FE is affecting the pathways to some extent, thus pointing out the different pathways in TP1 and TP2. The significant over-represented pathways were screened against 7 databases (Kyoto Encyclopedia of Genes and Genomes - KEGG, Edinburgh Human Metabolic Network - EHMN, Reactome, Integrating Network Objects with Hierarchies - INOH, HumanCyc, Biocarta, Pathway Interaction Database - PID) and selected (p ≤ 0.05) pathways were used for biological interpretations.

In Duroc, 32 metabolites were involved in 49 pathways in TP1 as compared to 35 pathways obtained by 70 unique metabolites in TP2 (Supplementary Table S4). Some of the underlying pathways in TP1 were the metabolism of glycerophospholipid, D-arginine and D-ornithine and choline; mTOR, Arf6, ErbB1, and Arf1 signaling pathways. However, in TP2, synthesis and degradation (Lysine, Valine-Leucine-Isoleucine, pyrimidine deoxyribonucleosides, methionine, glycine betaine, guanosine), bile salts and organic anion SLC transporter and pentose phosphate pathway were identified. Vitamin B6 metabolism was common between TP1 and TP2. Similarly, in Landrace, 36 unique metabolites from TP1 were involved with 20 significantly (p ≤ 0.05) over-represented pathways, while 37 metabolites were involved with 15 significantly over-represented pathways in TP2. Pathways like digestion of dietary lipid, synthesis of bile salts, valine degradation, valine-leucine-isoleucine biosynthesis were found in TP1. In TP2, the pathways found were degradation of pyrimidine deoxyribonucleosides, methionine, glycine betaine, cysteine biosynthesis, and vitamin B6 metabolism. However, the pathways involved were completely different in TP1 and TP2 in Landrace. This supports the fact that there is an observable difference in the biological level as shown by the difference in metabolites at two time points in both the breeds.

The complete breed analysis, combining both the time points, was also carried out to evaluate the differences in the metabolites and the biological pathways involved, that are specific to the breed. 101 unique metabolites were subjected to over-representation pathway analysis leading to their involvement with 50 pathways over-represented at combined time points in Duroc (Supplementary Table S4). Combining both the time points in Landrace, 66 unique metabolites pointed to 10 pathways that were significantly over-represented (p ≤ 0.05) (Supplementary Table S4). Biological oxidation, Histidine-lysine-phenylalanine-tyrosine-proline-tryptophan catabolism, and methionine salvage were involved with both Duroc and Landrace. All the other pathways were specific to each breed. The pathway differences between the breeds are also given in Supplementary Table S4.

Cluster analysis was carried out for the metabolites significant for RFI in a combined time point (Duroc – 36; Landrace – 29) (Table 1). The differences in the metabolite clustering for two time points in each breed is also observed in the heatmap (Figs. 2 and 3). Pathway analysis of the metabolites clustering together in the heatmaps is given in Supplementary Table S5. In Duroc, 4 significant clusters of 36 metabolites: Cluster 1 (metabo 3–14), cluster 2 (metabo – 16–13), cluster 3 (metabo 6–25), and cluster 4 (metabo 20–36) can be differentiated (Fig. 2). In Landrace, 4 significant clusters of 29 metabolites: cluster 1 (metabo 1 and 3), cluster 2 (metabo – 14–6), cluster 3 (metabo 13–21), and cluster 4 (metabo 19–17) can be differentiated (Fig. 3). The x-axis represented sample clustering of TP1 and TP2 in both the breeds. The annotation of the metabolites as given in the heatmap (y-axis) and their corresponding pathways are given in Supplementary Table S5.

### Network visualization

To visualize and interpret metabolomics data in the context of human metabolic networks, to trace connections between metabolites and genes, and to visualize compound networks, the unique metabolites were cross-referenced with the KEGG database. Only metabolites with specific KEGG IDs were considered for compound-gene network and pathway analysis. The hub metabolites were identified by taking the highly connected metabolites that were associated with more than one gene in the compound gene network (Supplementary Table S6). Hubs are the nodes that are more connected than the average or typical nodes, and consequently are more likely to play crucial biological role.

In Duroc, 63 genes in TP1 pointing to 6 hub metabolites were identified, while in TP2, 79 genes pointing to 14 hub metabolites were identified (Supplementary Table S6). The hub metabolites were specific for each time point. In Landrace, 87 genes underlying 9 hub metabolites in TP1, while 40 genes were pointing to 7 hub metabolites in TP2. 3-Methyl-2-oxobutanoic acid was common hub metabolite in Landrace TP1 and TP2 (Supplementary Table S6). S-(2,2-Dichloro-1-hydroxy)ethyl glutathione was a common hub metabolite identified in Duroc and Landrace TP1, while 3-Methyl-2-oxobutanoic acid and cholesterol sulfate was found to be a common hub between Duroc and Landrace TP2.

A combined time point analysis for the breed identified 20 metabolites for Duroc and 15 for Landrace. Choline, acetoacetate, (R)-Lactate, D-Erythrose 4-phosphate, 3,4-Dihydroxy-L-phenylalanine, Xanthine, Deoxyuridine, phenylacetaldehyde, pyridoxine phosphate, 4-Pyridoxate, Taurolithocholate sulfate, 5-Guanidino-2-oxopentanoate were specific for Duroc while L-Methionine, D-Glutamate, Thiamine, Deoxycytidine, Chenodeoxycholate were specific for Landrace.

**Figure 5:**
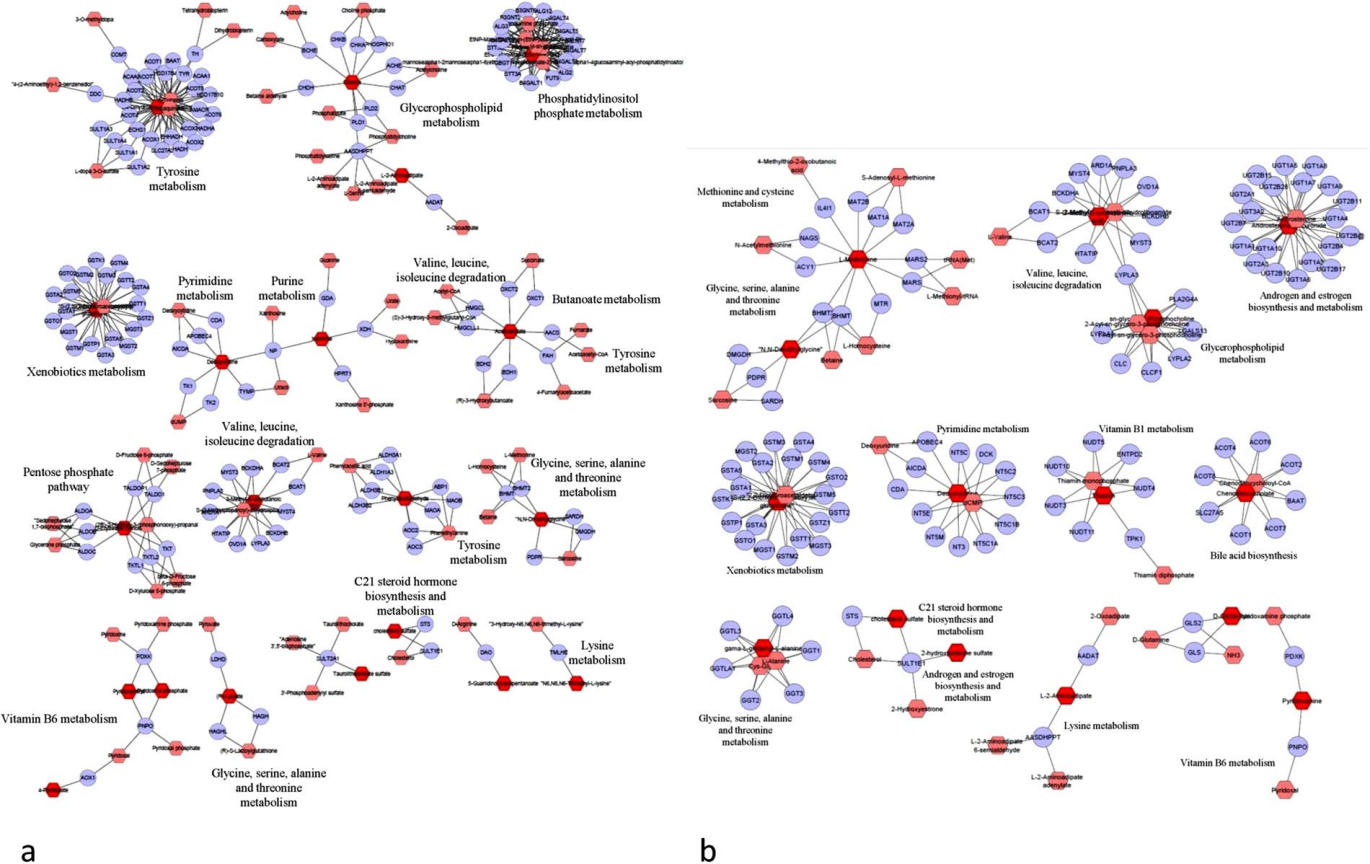
Compound-gene network for (**a**) Duroc (**b**) Landrace. The network is constructed using 101 metabolites underlying 17 different pathways in Duroc, while 28 metabolites are underlying 6 different pathways in Landrace.

Compound-gene network for both the breeds (Fig. 5) along with the putative genes (Supplementary Table S6) underlying the pathways were constructed. In Duroc, 32 metabolites were cross-referenced with the KEGG database thereby identifying 63 genes involved in 5 pathways in TP1 while 70 metabolites were related to 79 genes involved with 11 pathways in TP2. Glycerophospholipid and xenobiotics metabolism was specific pathways for TP1 after compound-gene cross-referencing while metabolism of butanoate, C21-steroid hormone biosynthesis, lysine, phosphatidylinositol phosphate, purine, pyrimidine pathways were specific at TP2. Metabolism of vitamin B6, tyrosine and Glycine-Serine-alanine-threonine was involved in both the time points (Fig. 5). In Landrace, 36 metabolites were related to 87 genes involved in 9 pathways in TP1 while 37 metabolites related to 40 genes involved with 5 pathways. Biosynthesis of androgen and estrogen, bile acid, C-21 steroid hormone. Metabolism of glycerophospholipids, methionine-cysteine, vitamin B1 and xenobiotics were specific for TP1 while metabolism of lysine, pyrimidine, vitamin B6 were specific for TP2. Glycine-Serine-alanine-threonine metabolism and valine-leucine-isoleucine degradation were common pathways between TP1 and TP2 in Landrace after cross-referencing of the metabolites. Interestingly, C21-steroid hormone biosynthesis and metabolism, glycerophospholipid and xenobiotics metabolism were identified only in TP1 in both the breeds and not present in TP2 while lysine, pyrimidine and vitamin B6 metabolism was identified only in TP2 in both Duroc and Landrace (Fig. 5).

## Discussion

Improving FE greatly reduces the feed expense and increases the profit for the producers. However, it difficult to measure as it involves the accurate recording of dry matter intake and other features^25^. Therefore, any reliable predictors of FE that can be easily measured and used in selecting animals would be helpful for pig producers. There are many genetic/genomic studies on pig FE in Danish pig breeds^9,26^. However, this is the first study to relate FE with metabolomics to identify metabolomic markers or signatures in Danish pigs.

In our study, using a high throughput UPLC/MS system, we analyzed metabolite concentration in blood collected before and after the FI testing period to search for a metabolomics signature with respect to the FE and other related traits in Danish production pigs at two time points. A clear clustering of sampling time and breed, among Duroc (TP2) and Landrace (TP1) (component 1) and Duroc (TP1) and Landrace (TP2) (component 2) gathered the samples according to their breeds and time points in four different groups, and supports the hypothesis of change in the metabolite profiles of the samples according to the breeds and time points. This also shows that the metabolite concentrations are not random and do have meaningful biological information.

We carried out an exploratory analysis by applying untargeted metabolomics, linear and network analyses, and pathway over-representation to unravel the effect of metabolites on FE phenotypes. A stronger association of metabolites with FE was expected at TP2; it is based on data recorded at the second sampling point. We do however believe that any metabolites found in TP1 would be more valuable for selection as this would allow for early screening of the pigs, leading to less wasted resources. As we also do find that the metabolites have a linear association between the time points, we do believe there is a potential for early screening using blood metabolites. Although the relation between the two time points and the lack of significant metabolites at TP1 may seem contradictory, it can likely be explained by several factors. The metabolite concentration in TP1 do not explain all the variation in TP2. If we combine this with the fact that FE is a multifaceted phenotype, which is not strongly controlled by a single factor, and in general is a somewhat subtle phenotype, it is easy to imagine that despite the connection between TP1 and TP2 we do not find the same results in both time points. Thus more data, and possibly a multiple-metabolite model may be needed for successful application of early screening.

From the KS test, we can observe that for most traits, the p-values are not uniformly distributed, with the highest p-value being 0.19. This means that if we apply FDR correction, all traits seem to have an overall relation to our traits, meaning even the borderline significant results are likely to be showing an underlying true effect. This establishes that metabolite profiles are a relevant source of information for our phenotypes of interest. Beyond the relevance of the metabolites for our phenotypes, we also established that for a large proportion of metabolites, the concentrations are linearly related over time. This shows us that despite variation over time, metabolites profiles show a level of temporal stability and predictability in our data.

From the heatmap clustering analysis of the top metabolites based on p-values in Duroc and Landrace separately for RFI, we observed that the samples clustered at two time points in both the breeds. A clear demarcation is observed while clustering the metabolites. In Duroc, the clusters identified were involved with the metabolism of phenylalanine, vitamin B6, arginine and ornithine, digestion of dietary lipids. Regarding Landrace, the clusters identified were found to be involved with biosynthesis of arginine-proline metabolism, bile secretion, and lysine degradation.

We applied a well-known gene co-expression network approach – WGCNA^17^ to analyze the metabolomics data in this study. From the network analysis, we found several modules associated with FE, TDG, and RFI in both the breeds at different time points, pointing towards the common pathways influencing these traits. The change in the metabolites found at different time points supports the fact that there are changes in metabolomic levels related to FE, TDG, and RFI between Duroc and Landrace breeds. We also constructed a compound-gene network for the significant unique metabolites in Duroc and Landrace to identify the pathways after cross-referencing with the KEGG pathways specific for humans and identifying genes underlying these pathways.

Based on the over-representation pathway analysis, we identified some key pathways in two time points in each breed (Supplementary Table S4). We also created a compound-gene network by applying Metscape 3.1.3. The compound-gene network in Duroc pointed towards 13 specific pathways underlying metabolism (butanoate, glycerophospholipid, glycine-serine-alanine-threonine, lysine, purine, and pyrimidine, vitamin B6, tyrosine, C21-steroid hormone, phosphatidylinositol phosphate, and xenobiotics), valine-leucine-isoleucine degradation and pentose phosphate pathway (Fig. 5a). In Landrace, 12 pathways were identified with some of them overlapping with Duroc, while androgen and estrogen biosynthesis, bile acid biosynthesis, methionine, and cysteine metabolism and Vitamin B1 metabolism specific to Landrace (Fig. 5b).

Among all the key metabolites identified in Duroc TP1, we identified choline (C00114), which is involved in glycerophospholipid metabolism and glycine-serine-alanine-threonine metabolism. Choline was found to be a hub metabolite involved with both FE and RFI in Duroc TP1 (Fig. 5, Supplementary Table S3). Choline is an essential nutrient for normal animal growth and performance and has been used as a supplement in the animal diets. Being an essential component of the cell wall and fat metabolism, choline is found to enhance FE and weight gain in ruminants^27^. Furthermore, Choline is a methyl donor taking part in DNA methylation, and is a vital process control the correct expression of genes thus ensuring proper cell development and growth^28^.

The hub metabolite pyridoxamine (C00534), was found to be significant in Duroc TP1 (RFI) and Landrace TP2 (EDG, DG), which was identified for Vitamin B6 metabolism. Pyridoxamine phosphate plays an essential role in the interaction of amino acid, carbohydrate, fatty acid metabolism, and TCA cycle. Studies reported the relationship of B6 in tryptophan metabolism of weanling piglets but were unable to detect an effect on the oxidation of the tryptophan pathway and suggested that B6 may stimulate another pathway in tryptophan metabolism^29^. Metabolic shifts in lipid and carbohydrate utilization in high FE animals were reported^14^. They also reported reduced hepatic usage of fatty acid in high FE animals with a molecular alteration in lipid metabolism. A complementary analysis pointed out increased circulating triglycerides accompanied by a lower concentration of saturated and polyunsaturated fatty acids in the liver of high FE pigs^14^.

We identified acetoacetate (C00164) to be the most significant for pathways underlying metabolism of butanoate, tyrosine, and valine-leucine-isoleucine degradation in Duroc TP2. Since butanoate is a metabolite of gut flora and involved with energy metabolism, butanoate metabolism may be activated under the conditions of cellular stress^30^. Oxidative stress reprograms lipid metabolism increasing the mitochondrial fatty acid oxidation^31^. Butanoate metabolism was also found to be enriched for differentially expressed genes in Nelore cattle muscle for RFI^32^. In our study, we found acetoacetate as the hub metabolite responsible for butanoate metabolism related to TDG in Duroc TP2 (Supplementary Table S3). However, in the study reported by Akbar^33^, the subcutaneous administration of acetoacetate did not affect the FI. Acetoacetate was also found responsible for tyrosine metabolism as it affects *FAH*, an enzyme that catalyzes the last step of tyrosine metabolism. Metatranscriptomic studies revealed the tyrosine pathway to be differentially expressed in rumen microbiome of beef cattle^34^. Acetoacetate was also found as the hub (Fig. 5) for valine, leucine, and isoleucine pathway. We identified 3-methyl-2-oxobutanoic acid specific for this pathway also found to be associated with Duroc TP2. The three amino acids in the pathway are essential and act as a building block for tissue protein synthesis^35^.

Deoxyuridine (C00526) and xanthine (C00385) were found to be involved with purine-pyrimidine metabolism in Duroc TP2. Deoxyuridine was associated with FE, while xanthine was associated with TDG in Duroc TP2 (Supplementary Table S3). Both the metabolites are involved in pyrimidine metabolism and are part of the cecal content of digestive segments involved with direct or indirect synthesis or utilization of compounds by the gut microbiota^36^. These metabolites were also reported to be affecting the digestive efficiency in chickens^37^. Previous studies have shown an increase in the concentration of xanthine by increased FI^38,39^. The degradation of rumen fluid into xanthine, hypoxanthine, and uracil by the action of bacterial nucleic acids (DNA, RNA) was reported previously^40^. The decrease in the rumen pH in dairy cows fed with high-grain diets changes the microbiota composition due to their intolerance towards low pH^41^.

We identified cholesterol sulfate (C18043) associated with FE, TDG, and RFI in Duroc TP2, whereas with RFI in Landrace TP1 (Supplementary Table S3). The relationship among FI behavior, cholesterol, and triglyceride plasma levels in pigs was reported by Rauw *et al*.^42^, wherein a strong co-relation between FI and cholesterol levels was established. However, these authors reported a weak correlation between RFI and cholesterol levels that were completely insignificant after correcting for the FC. The cholesterol pathways were also found to be consistent with the study involved in the regulation of FE in cattle (Holstein and Jersey) as reported by Salleh *et al*.^43^.

Pathways such as lysine metabolism are affected by the metabolite 2-Aminoadipate (C00956) and were related to TDG in Duroc TP2, EDG and DG in Landrace TP2, respectively (Supplementary Table S3). Lysine is a limiting amino acid, and its deficiency impairs the animal’s immunity and growth performance^44^. Yin *et al*.^45^ suggested that the dietary supplementation with lysine influences intestinal absorption and metabolism of amino acids. Lysine restriction inhibits intestinal lysine transport and promotes FI associated with gut microbiome in piglets^45^.

Functional annotation revealed some pathways involved with the metabolism and digestive gland secretion during feeding over-represented among the unique hubs and their role in FE, EDG, TDG, DG, and RFI in pigs. Based on the potential role of these metabolites in the metabolism of carbohydrate (butanoate), lipid (steroid, glycerophospholipid, pentose phosphate pathway, bile acid), amino acid (Gly-Ser-Ala-Thr, Lysine, Methionine-cysteine, tryptophan, tyrosine, valine-leucine-isoleucine), nucleotide metabolism (purine, pyrimidine), metabolism of cofactors and vitamins (B3, B6), and metabolism of xenobiotics, their involvement in the feeding behavior and FE traits are conceivable.

The genes identified from the compound-gene network were checked against the previously identified QTLs obtained from Animal Genome PigQTL database, where all previous research on QTLs is curated. Among the 198 genes identified for both Duroc and Landrace from both the time points, 9 genes were previously reported as candidate genes in the QTL database with varied traits (Supplementary Table S6). *NT5E* associated to Deoxycytidine in Landrace TP2 was identified as a candidate gene for RFI in the QTL database^46^. *HSD17B4*, which was associated to 3,4-Dihydroxy-L-phenylalanine in Duroc TP1, was identified as a candidate gene for carcass weight, backfat at tenth rib, and drip loss in Berkshire pigs^47^. Previous studies show the relation of FE with pork quality. Some studies reported that animals with low RFI have less back fat^48–50^, less water holding capacity^48^ and impaired sensory quality^50^. However, in some other studies, no difference was observed in the pork quality from low RFI pigs and controls with respect to drip loss, but a correlation between RFI and sensory traits related to reduced intramuscular lipid was observed^51^. A candidate gene, *MAOA*, associated to phenylacetaldehyde, was identified in our study that has been reported previously for intramuscular fat, ADG, and loin muscle^52^. Previous studies in Duroc reported high genetic variability due to moderate to high heritabilities for RFI, growth and carcass traits. An increase in the loin eye area was reported with decreased RFI, backfat and intramuscular fat content in Duroc pigs^53^. *NUDT3* was related to thiamin in Landrace TP1 and *PLD2* related to choline in Duroc TP1 in our study was also found to be a candidate gene for loin muscle area and loin muscle depth in pigs^54,55^. The metabolites and the genes identified are consistent with FE related traits. Further studies are warranted to evaluate the repeatability of our results in other pig population.

## Conclusions

Our integrated approach using data annotation, linear model association, weighted metabolite network analysis, and pathway over-representation analysis indicated potential targets for biological processes related to FE. The significant metabolites affecting the pathways points out the role of the metabolites concerning to FE and related traits. Overall, we observe several trends in the results. We are able to identify relevant biological relation between our traits and metabolite profiles, but also differences in breed and time points. In contrast, we also see that there is some linear predictability in the metabolites between time points. As the pigs are entering and undergoing a very rapid growth and maturation rate between samplings, it is natural to expect that the underlying metabolite profiles and networks are changing, despite elements of stability in metabolite profiles. This means that strategies for applying metabolite information into a real life farming appear to be complex and require good understanding of the relations and changes in metabolite profiles and time, and the identification of not only key metabolites, but also key time points. Validation of these results in a cohort with more animals and time points would help to establish a framework for future FE prediction using metabolomics biomarker profiles that could be practical to use in large populations other than genomic profiling. More data would also make it possible to model the complex relations in metabolite profiles over time more accurately. Further understanding of the mechanisms driving these trends will result in improved nutrient utilization, reduction in production costs, and increased FE in pigs. To best of our knowledge, this is the first study to report metabolomics profiles related to FE and related traits in Danish pigs.

## Methods

### Ethical approval

The blood sampling and experiment were approved and carried out in accordance with the Ministry of Environment and Food of Denmark, Animal Experiments Inspectorate under the license number (tilladelsesnummer) 2016-15-0201-01123, and C-permit granted to the principal investigator/senior author (HNK).

### Study design and phenotypes

The pigs used in this experiment were housed at the pig testing station “Bøgildgård” operated by SEGES within Landbrug and Fødevarer (L&F: Danish Agriculture and Food Council). Pigs were *ad libitum* fed and free water supply. The authors of this study were not responsible for animal husbandry, diet, and care as the testing station is a facility within the Danish breeding program, run by SEGES.

Blood samples were collected at a boar testing station Bøgildgård, owned by SEGES. The pigs were purebred uncastrated males from Danbred Duroc (n = 59) and Danbred Landrace breeds (n = 50), amounting to a total of 109 pigs. The initial bodyweight of the pigs before the testing period was approximately 7 kg, followed by a 5-week acclimatization phase. The pig diet consisted of a feed mixture with the main ingredients being: 39% barley, 27% wheat, 14% soybean meal and 6% oats. For the phenotypic traits, the weight of FC in kg and FE for each pig in the testing phase was measured beginning with an initial weight of around 28 kg for each pig. Bodyweight measured at two time points, the beginning and end of the test, were available from standard test procedure of the testing station and their difference was referred to as delta weight (DW). FE was calculated as the ratio between DW and FC. The testing phase ranged from 41 to 70 days based on the viability of each pig. The DG was calculated for three time phases – birth to testing (EDG), testing start to end (TDG), and birth to testing end (DG). RFI was computed as the difference between the observed daily feed intake (DFI) and the predicted feed intake (pDFI)^56^. All pigs consumed the same feed until the test end.

For the study of metabolites, approximately 5 mL of blood was collected from jugular venipuncture from each pig into tubes containing ethylenediaminetetraacetic acid (EDTA) and immediately placed on ice. Samples were collected at two time points, one at the start of this test phase (approximately 28 kg weight) and the second after 45 days referred to as TP1 and TP2 in the further study. The pigs were sampled at the same time of the day and same day of the week to insure the most comparable sampling. Pigs were in non-fasted state. For the separation of the blood plasma, samples were centrifuged at 3000 g for 10 minutes at 4 °C, and plasma was stored at −80 °C.

### Non-targeted metabolomics analysis

The plasma samples extracted from each pig were subjected to metabolomics analysis. The samples were processed by MS-Omics (http://www.msomics.com/; Denmark), and the analysis was carried out using a UPLC system (UPLC Acquity, Waters) coupled with time of flight mass spectrometer (Xevo G2 Tof Waters). An electrospray ionization interface was used as an ionization source. The analysis was performed in negative and positive ionization mode. The UPLC was performed using a slightly modified version of the protocol described by Catalin *et al*. (UPLC/MS Monitoring of Water-Soluble Vitamin Bs in Cell Culture Media in Minutes, Water Application note 2011, 720004042en).

Raw files were processed using MZmine 2^57^. The mass detection was ascertained, keeping the noise level at 1E2 (negative mode) and 1E3 (positive mode). The chromatogram building was achieved using a minimum time of 0.05 min, a minimum height of 1E3 (positive mode) and 4E2 (negative mode), and *m*/*z* tolerance of 0.01 (5ppm). The local minimum search deconvolution algorithm was used with a baseline cutoff, the minimum peak height of 2E3 (positive mode) and 5E2 (negative mode), and peak duration range of 0.04–5.0 min (positive mode) and 0.05–5.0 min (negative mode). Chromatograms were deisotoped with *m*/*z* tolerance of 0.01 (or 5 ppm) and an RT tolerance of 0.2 minutes for positive and 0.5 minutes for negative modes, respectively. Peak alignment was performed with (*m*/*z* tolerance at 0.01 (or 5 ppm). The peak list was eventually gap-filled with the peak finder module (intensity tolerance at 50% and *m*/*z* tolerance at 0.01 (or 5 ppm).

The identification of the metabolites was performed using both peak retention times (compared against authentic standards included in the analytical sequence) and accurate mass (with an acceptable deviation of 0.005 Da). As a standard quality control, samples with blank >3 were not included. The relative standard deviation between QC samples was kept less than 60, the correlation between the dilution of QC and response was >0.8.

The data were aligned and normalized using total ion intensity. The metabolites were identified by comparison with the online Human Metabolome Database (HMDB)^58^ using exact *m/z* values and retention time. The metabolites that did not correspond to HMDB were left unannotated. These compounds were annotated based on a library search of the masses in the HMDB with a mass uncertainty of 0.005 Da or 5 ppm. The search in HMDB assumes that all ions originated from the [M + H]^+^ or [M + Na]^+^ (in positive ionization) or [M − H]^−^ (in negative ionization) ions.

### Metabolite-trait association analyses

The metabolite data were log-normalized before fitting the linear model. For each group of metabolites, only those with relative standard deviation >0.15 were used, based on the raw counts. The log-normalized metabolite concentration (*m*_*ijk*_) was adjusted for fixed and random effects as follows.

1 $${m}_{ijk}={B}_{i}+{S}_{j}+{P}_{k}+{\varepsilon }_{ijk}$$

where,

*m*_*ijk*_: is the relative concentration of each metabolite;

*B*_*i*_: is the fixed effect for the breed;

*S*_*j*_: is the batch effect;

*P*_*k*_: is the random effect from the pen;

*ε*_*ijk*_: is the random residual effect associated with each observation.

For each adjusted metabolite, denoted as $\widehat{{m}_{ijk}},$(where, $\widehat{{m}_{ijk}}=({m}_{ijk}-{\hat{B}}_{i}+{\hat{S}}_{j}+{\hat{P}}_{k}$) from Eq. 1), the linear association with the pig phenotypes was estimated based on the following model:

$${y}_{ij}=\widehat{{m}_{ijk}}+{A}_{j}$$

Where,

*y*_*ij*_: is the phenotype (FE, EDG, TDG, DG, RFI) for each animal;

$\widehat{{m}_{ijk}}$: are the adjusted metabolites based on the Eq. (1);

*A*_*j*_: is the covariate for animal’s sampling age in days;

We did not include the sampling age with our other fixed and random effects when correcting our metabolites, as the sampling age is correlated with our phenotypes. This is because the slower-growing pigs have a higher sampling age as it takes long time for them to reach the testing phase. Adjusting for sampling age *a priori* would thus create biases^59^. Thus, we included sampling age as a covariate in the final models associating corrected metabolites with our phenotypes.

Many models were used, so instead of looking into the specific results of each metabolite in each model, we initially tested the significance of the model based on all metabolites. This was done by using the Kolmogorov-Smirnov test to compare the resulting p-value distribution with the uniform distribution for the parameter of interest in each batch. Cluster analysis and heatmap of significantly different metabolites were generated using the ‘pheatmap’ package in R (v1.0.12).

### Metabolite network analysis

Network analysis was performed using Weighted Gene Co-expression Network Analysis (WGCNA) R package version 1.66^17^. The WGCNA methods have been successfully applied to gene expression data from microarrays^60^ and RNA sequencing platforms in animal sciences^18^. Recently this methodology was applied on genome-wide genotype data as well^61^. Hereby, we extended this methodology to build networks using metabolomics data. The methodology, in summary, involved the Spearman correlation between all adjusted metabolite concentrations followed by the transformation of the correlation matrix into an adjacency matrix (AM) by fitting a power coefficient beta (β). The β was chosen by testing the coefficient between 12 and 22 and selecting the one that maximizes the scale-free topology based on the scale-free R^2^ value >0.8. From the scaled correlation, the Topological Overlap Measure (TOM), representing the connection between metabolites was calculated. Based on the TOM and applying the *dynamicTreeCut* algorithm, modules of connected metabolites were generated. In each module, the eigengene values of the module metabolites were calculated.

A linear model was fitted among the eigengene values of metabolite modules and the phenotypes to assess the module-phenotype relationship. Further, we intersected the metabolites identified based on the linear association with those from the modules significantly associated with phenotypes. Metabolites with p-values ≤ 0.05 and those clustered into the modules with a phenotypic correlation ≥0.2 and p ≤ 0.1 were selected for subsequent analysis.

### Pathway analysis and network visualization

Over-representation analysis was performed using IMPaLA^24^ to identify metabolites underlying pathways meaningful to FE related-traits. IMPaLA takes into account the pathways from 11 public databases, including Reactome^62^ and KEGG pathway^63^. Over-represented biological pathways were taken as significant with p ≤ 0.05.

The visualization of metabolomic data was done in the context of human metabolic networks using Metscape v 3.1.3^64^, a Cytoscape plugin. Based on that, we identified the connections between metabolites and the putative genes underlying the pathways in a compound-gene network approach. The key metabolites that were found to be involved in the main pathways were referred to as hub metabolites. The hub metabolites (compound IDs) from each significant pathway were selected and visualized using Cytoscape. A schematic representation of the methodology is given in Supplementary Fig. S3.

## Supplementary information

Supplementary figures.

Dataset 1.

Dataset 2.

Dataset 3.

Dataset 4.

Dataset 5.

Dataset 6.

## Data Availability

The datasets generated and/or analyzed during the current study are publicly available upon acceptance of the paper at Metabolights database https://www.ebi.ac.uk/metabolights/MTBLS1384 with accession ID: MTBLS1384. 10.1093/nar/gks1004. PubMed PMID: 2310955.
