# Supplementary material for: Metabolomic networks and pathways associated with feed efficiency and related-traits in Duroc and Landrace pigs
Source: Sci Rep. 2020 Jan 14;10:255. doi: 10.1038/s41598-019-57182-4 (PMC6959238; doi:10.1038/s41598-019-57182-4)
Supplement: Supplementary file 1 — Supplementary figures. [file 41598_2019_57182_MOESM1_ESM.docx]

**Metabolomic networks and pathways associated with feed efficiency and related-traits in Duroc and Landrace pigs**

Victor Adriano Okstoft Carmelo^1^, Priyanka Banerjee^1^, Wellison Jarles da Silva Diniz^1, 2^ and

Haja N. Kadarmideen^1*^


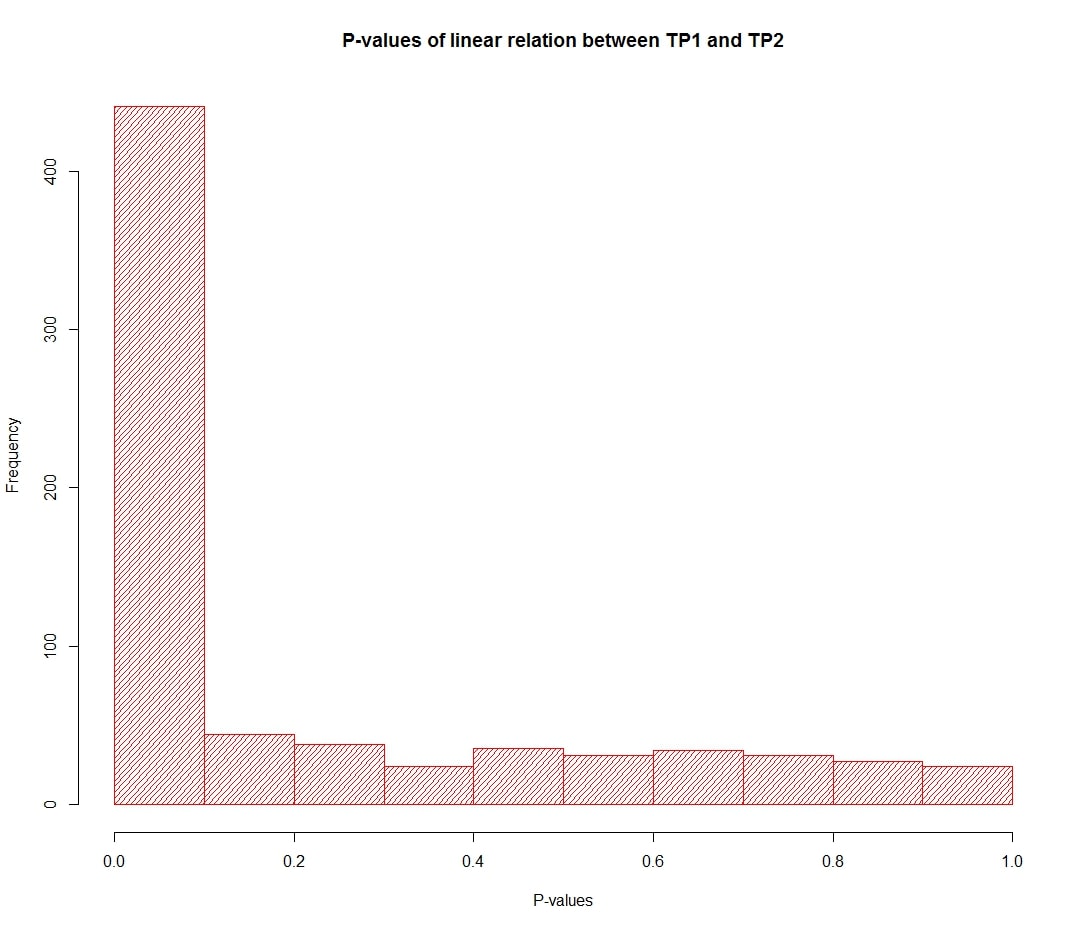
 Supplementary Fig. S1: P-value for the linear relationship between TP1 and TP2 (Duroc and Landrace combined) for each metabolite. The metabolites were log-transformed. The model fit is a simple linear regression only including an intercept, the metabolite values from the first sampling and the metabolite from the second sampling as a responsive value. No other covariates are needed since each metabolite is paired between each pig.


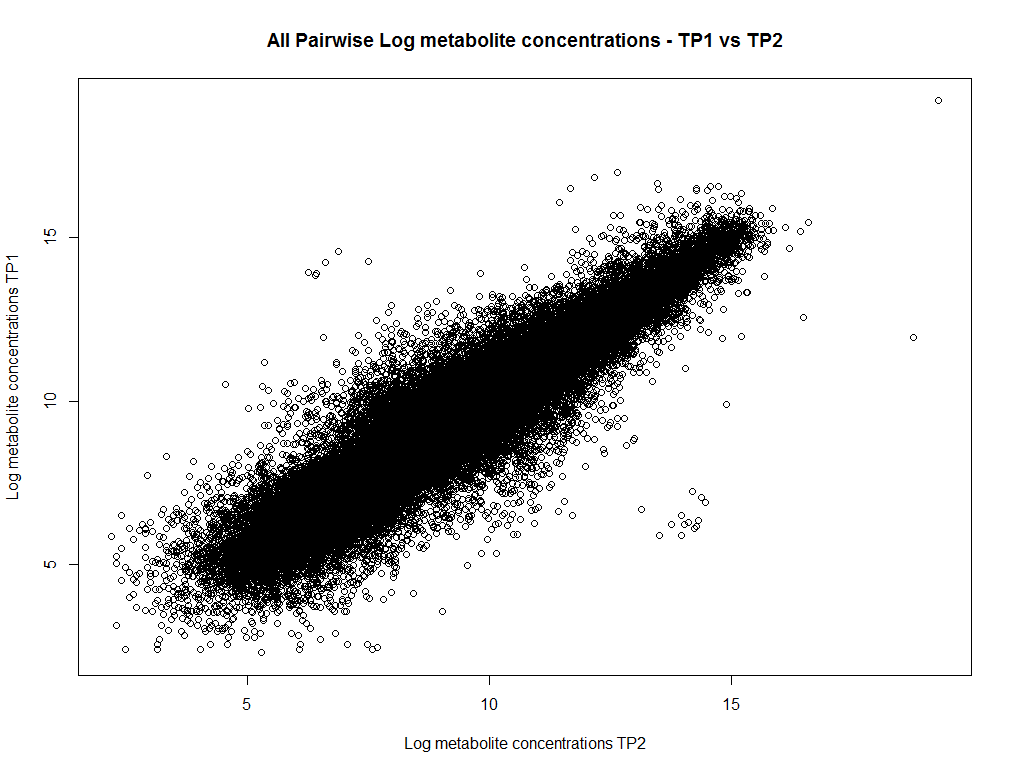


Supplementary Fig. S2: All pairwise metabolite concentrations between TP1 and TP2 for all metabolites and all pigs. We see that there is a strong linear relationship between the metabolite concentrations in the two timepoints, with a Pearson correlation of 0.935.
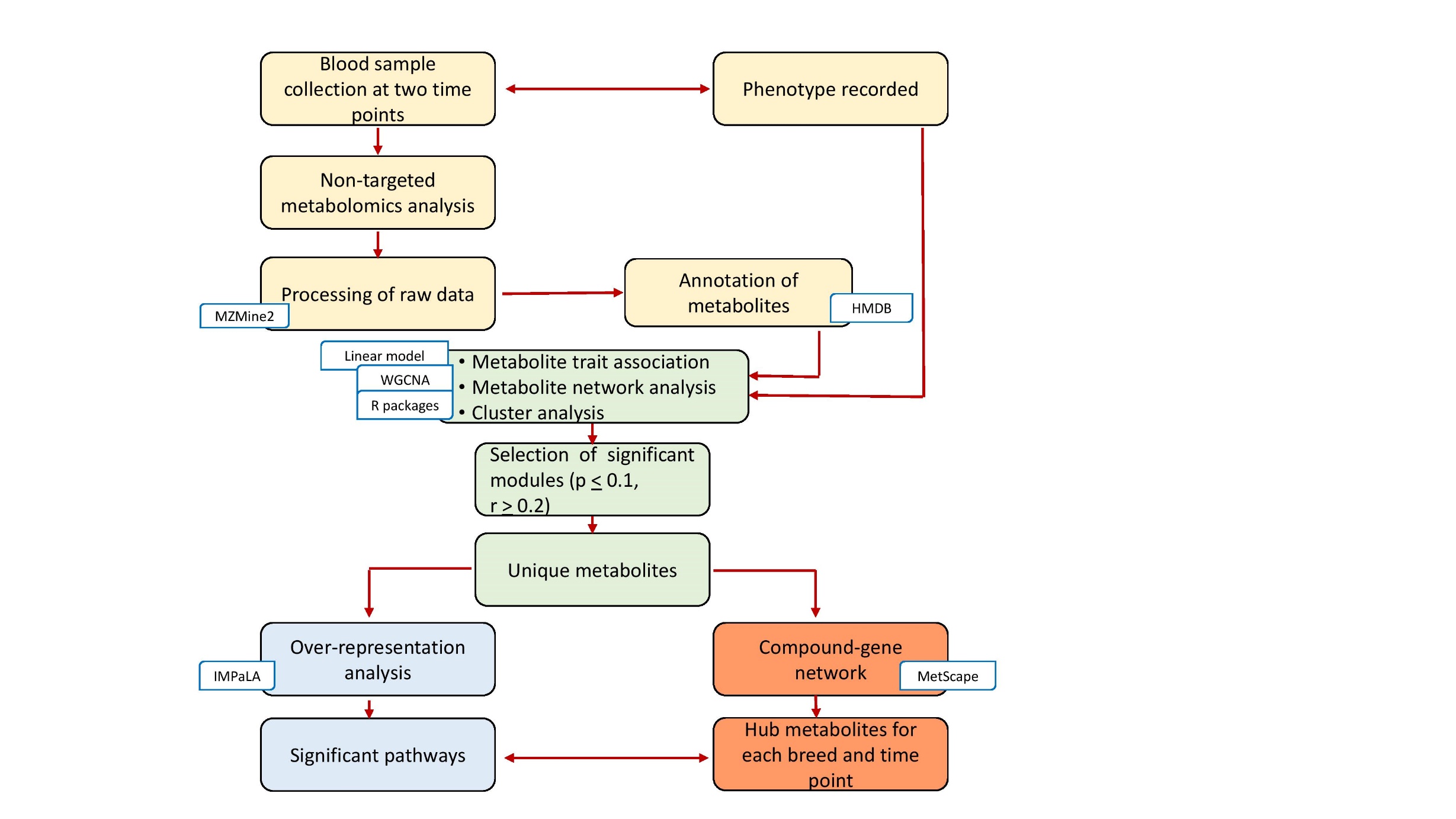


Supplementary Fig. S3: Schematic workflow for identification of hub metabolites for each time point in Duroc and Landrace.
